# A study on reintegration of street children in Burundi: experienced violence and maltreatment are associated with mental health impairments and impeded educational progress

**DOI:** 10.3389/fpsyg.2014.01441

**Published:** 2014-12-16

**Authors:** Anselm Crombach, Manassé Bambonyé, Thomas Elbert

**Affiliations:** ^1^Department of Psychology, Clinical Psychology and Clinical Neuropsychology, University of KonstanzKonstanz, Germany; ^2^Department of Clinical Psychology, University Lumière of BujumburaBujumbura, Burundi; ^3^Non Governmental Organization Vivo InternationalKonstanz, Germany

**Keywords:** Burundi, street children, residential centers, posttraumatic stress disorder, maltreatment and success in school, reintegration

## Abstract

Street children are exposed to violence, and subsist in poor and generally precarious conditions. In conflict regions, institutional care facilities are often the only well established way to care for vulnerable children. Providing access to school education is considered to be key to allow successful integration into society. However, adverse effects of psychological disorders may pose another serious obstacle. In semi-structured interviews in a sample of 112 Burundian male youths (mean age = 15.9 years), we assessed exposure to traumatic stressors, regularly and recently occurring violence as well as prevalence of posttraumatic stress disorder (PTSD), depression, substance dependence, suicidal risk, and progress in school. Former street children (*n* = 32) and other vulnerable children (*n* = 50) in a residential center were compared to children living in the streets (*n* = 15) or with families (*n* = 15). While the children living in the center were less regularly exposed to violence and reported less substance dependence than street children, PTSD symptoms were common among the former street children. Furthermore, we provided empirical evidence that for the children living in the center, recently experienced violence – mostly minor physical conflicts, psychological violence and neglect – was associated with increased PTSD symptomatology and impeded progress in school. In a population of children who experienced many traumatic incidences and a lot of violence, even minor violent events may trigger and reinforce PTSD symptoms. Hence controlling exposure to violence and addressing mental ill-health in vulnerable children is mandatory for reintegration.

## INTRODUCTION

“*Street children [...] are [...] among the most invisible and, therefore, hardest children to reach with vital services, such as education and health care, and the most difficult to protect.*”

(United Nations Children’s Fund, 2006, p. 40)

Street children lives are marked by frequent, and in some cases continuous exposure to violence. They have typically already suffered abuse at home in dysfunctional families, and have grown up in poverty-aﬄicted, chaotic neighborhoods, experiencing both violence in the streets, and mistreatment by police forces (Le Roux, 1996; Ochola, 1996; Scanlon et al., 1998; Kidd, 2003; Gaetz, 2004; Young, 2004; Pinheiro, 2006; Thomas de Benitez, 2007). These violent experiences, combined with the constant insecurity of having to struggle to find food and shelter, put these children at substantial risk of developing trauma-related mental disorders (Veale and Dona, 2003; Turnera et al., 2006; McManus and Thompson, 2008; Cleverleya and Kidd, 2011). Several studies suggest that children living in the streets frequently suffer from behavioral and emotional difficulties, such as low-self-esteem, suicidal ideation or even suicidality, alcohol and substance abuse, depression and posttraumatic stress disorder (PTSD; Greene et al., 1997; Tyler et al., 2003; Stewart et al., 2004; Thompson et al., 2006; Ahmadkhaniha et al., 2007; Jones et al., 2007; Kerfoot et al., 2007; Kidd and Carroll, 2007).

It can be assumed that the risk of developing trauma-related mental ill-health is even higher for these children and youths in crisis and war regions, as studies have shown PTSD prevalence rates between 20 and 50% for war-affected children in conflict regions such as Bosnia, Sri Lanka and Rwanda, even years after the exposure to war (Smith et al., 2002; Schaal and Elbert, 2006; Elbert et al., 2009). A greater exposure to traumatic stressors is associated with a higher probability of suffering from PTSD (e.g., Neuner et al., 2004). Schauer et al. (2005, 2011) detailed a theoretical approach of understanding this phenomenon: Whenever individuals experience traumatic events fear conditioning connects perceptive memory elements to trauma-related cognitions, emotions and physical responding, thereby creating an interconnected associative memory representation, called a *fear* network. With increasing exposure to traumatic stressors, this associative network becomes detached from the contextual information – the when and where events had happened – for two reasons: first, the extreme state of emotional arousal during a traumatic event affects hippocampal functioning, which could explain the reduced consolidation of declarative memories in PTSD (Elzinga and Bremner, 2002). Second, the perceptual-cognitive-emotional associations for several traumatic experiences merge in one associative network. This makes it increasingly difficult to disentangle the different traumatic experiences, and particularly, to recall the adequate corresponding contextual information. In consequence, the fear/trauma network cues strong feelings of a present danger, helplessness, insecurity, and fear within the individual. The ignition of just a few elements in the network may be sufficient to activate the whole structure (e.g., Schauer et al., 2011). We postulate that the persistent insecure and violent situations that children living in the streets and other vulnerable children are exposed to easily trigger and enlarge the fear-network, reinforcing PTSD and other trauma-related disorders. However there is a dearth of studies assessing the mental health of street children in crisis and conflict regions.

Institutional care facilities have been put into place to respond to the needs of orphaned and other vulnerable children in countries affected by civil war, i.e., to support them with nutrition, places to sleep, medical care, access to education, and psychosocial support (Williamson and Greenberg, 2010). The principal objective is the reintegration of these children into society, either by enabling them to return to their families or by helping them to start an independent life. School education in particular is considered to be the key element for a successful reintegration of vulnerable children because it enhances the chances of living a healthy as well as financially and socially secure life (United Nations Children’s Fund, 2006; Betancourt et al., 2008). The benefits of a good school education are so well-known that sometimes even children leave their families or are sent by their parents to institutional care facilities to give them access to education, with the hope that they will eventually overcome their poor living conditions (Uvin, 2009; Williamson and Greenberg, 2010).

However, keeping the high risk of street and other vulnerable children for mental disorders in mind, providing nutrition, sleeping places and access to education may not be sufficient for the children and youths to successfully integrate into civil society. In fact, it is known that institutional care can have detrimental effects on the development of children and youths. This is exacerbated in institutional situations where children accustomed to violence are packed into overcrowded, poorly conditioned, under-managed and under-staffed conditions. These factors can perpetuate a violent and abusive atmosphere and thereby expose the children to other forms of insecurity, neglect and deprivation (Thomas de Benitez, 2007). Additionally, caretakers in the institutional facilities are often not trained to deal with these children and are accustomed to using corporal punishment and threats in order to establish their authority (Gershoff, 2002; Benjet, 2010; Straus, 2010). Such behavior however reinforces and maintains the behavioral and emotional problems of the children and youths (Cyr et al., 2006; Greenfield and Marks, 2010; O’Donnell et al., 2011) and endangers the development of a reliable, emotionally safe connection. This vicious cycle is compounded by the fact that these children have already experienced violence and abuse within their families of origin, neighborhoods and school (Ramphele, 1997; Benjet, 2010). On the other hand, institutional care facilities may provide the children with a more stable and secure environment than the privations and dangers of the streets, thereby improving their mental health.

Recent studies suggest that exposure to community violence, i.e., witnessing or experiencing potentially traumatic incidences, or even peer victimization, strongly impairs academic success in school by amplifying symptoms of depression and PTSD (Schwartz et al., 2005; Mathews et al., 2009). This would be particularly true for children who have previously been exposed to traumatic experiences and insecure living conditions. Hence, institutional care facilities might undermine their own foundation for successful reintegration of the children, should they fail to provide a safe, predictable and violence free environment.

In this study we examined current and former street children in Burundi. To account for the diverse life stories of the children in Burundi, we defined street children as all those who were exposed to the dangers of the streets and who had to struggle to survive on their own. Burundi, with a population of more than 10 million inhabitants, is one of the smallest and most densely populated countries within the Great Lakes region of Eastern Africa, a region that has been destabilized severely by many wars and conflicts over the last 40 years (Hatzfeld, 2004; Johnson, 2008; Pham et al., 2009). Burundi’s history over this time epitomizes this, with a long simmering conflict escalating intensively in 1993, and lasting until 2006. During “la crise” – as the Burundians refer often to this war – over 300,000 persons were killed, more than 500,000 had to flee and over 800,000 were internally displaced (Uvin, 2009). Today the population still suffers from its consequences in the form of poverty, and family conflicts and has to deal with an atmosphere of violence and insecurity created by politically motivated killings (Human Rights Watch, 2012). A significant portion of the population lacks a stable food supply. Moreover, a myriad of conflicts relating to private property rights troubles many families. Lands are divided so that the resulting small plots are insufficient to sustain their owners (Watt, 2008; Armstrong, 2011).

In 2011, the International Rescue Committee, the United Nations Children’s Fund and the Burundian Ministry of National Solidarity, Human Rights, and Gender published a report on the situation of children in residential centers in Burundi. The analysis encompassed 98 centers in total, in which 5,520 children were taken care of. However, only few centers care for extremely vulnerable children who have been living on the streets. In order to assess our hypothesis, we chose a center run by a local NGO in Bujumbura, the capital of Burundi, for evaluation. This center took care of boys only, and included children and youths who had lived on the streets. The annual budget per child was approximately 550,000 fbu (≈314 €), which is higher than the average rate for this country (≈180 €; Armstrong, 2011).

With this study we aimed to assess the extent to which violence can affect mental well-being and psychosocial functioning and thus limit the possibility for reintegration. We hypothesized that the children with experience of life on the street or other dangerous experiences would suffer more from psychological disorders than children without such a history. However, we also predicted that they would suffer less than children who were still living in the streets. Furthermore, we predicted that orphaned and vulnerable children were more affected than children who had grown up with their families and still lived in Bujumbura with them. Finally, we expected that even minor violent experiences trigger and reinforce PTSD symptoms impairing progress in school on the long run. In addition the impact of time spent within a center on the mental well-being and functioning of the children was explored.

## MATERIALS AND METHODS

### PARTICIPANTS AND LIVING CONDITIONS

All boys (*n* = 82) living in the center in 2011 were examined using structured clinical interviews. The age ranged from 10 to 23 years. All children went to school or received vocational training. Furthermore they had a place to sleep, received food twice a day and had access to running water and sanitary facilities. On weekdays two male and two female educators took care of the children. The director of the center at that time was a nurse, who stayed overnight and on holidays in the center. Additionally one educator looked after the children on the weekend. A Burundian psychologist provided psychosocial support in the form of individual counseling when specific problems emerged or needed to be addressed. Additionally two cooks and two guards worked in the center and sometimes psychology and education students provided extra assistance. One child was excluded from the analysis due to a neurological disorder (epilepsy, treated with carbamazepine).

Fifteen additional boys living in the streets at the time of the investigation and 15 more who were living in families in Bujumbura also participated in the study. Groups were selected so that they had a comparable age range. In order to obtain a representative sample, three different places in Bujumbura were selected to contact children living in streets. Children there were randomly invited to participate in the study. The interviews were conducted in private on the premises of the Red Cross Burundi. The families invited to participate were also chosen randomly. The quarters, streets and houses were approached in a random order to recruit boys within the given age range. One child was selected from each quarter, in order to attain a representative sample for the city of Bujumbura. Children living in families, who had reported street experience (*n* = 2) and with mental disabilities (*n* = 1) were excluded from further analysis. The interviews were conducted privately in the family homes.

Thirty-two of the children living within the center were considered as belonging to a very high-risk population for mental disorders because they had spent part of their lives on the streets and had been potentially exposed to very difficult living conditions. These *former street children* were compared to the *former family children* living in the center, current *street children* and current *family children*. The Ethical Review board of the University of Konstanz approved the study based on the Declaration of Helsinki and the University Lumière of Bujumbura assisted with the implementation. All participants gave their informed consent. For participants under the age of 18 the legal guardians gave informed consent, if available. While boys in the center profited later from restructuring of the center, therapies (see Crombach and Elbert, 2014a) and other supporting activities, the children in the streets and in the families received a financial compensation of 5,000 fbu (≈2,86 €).

### PROCEDURE

The assessment was conducted from January to April 2011 in the center and between March 2011 and June 2011 on the streets and within the families. The principal investigator of this study (who lived in Bujumbura before and during the period of the assessment) and another psychologist with clinical training and work experience in Germany and East Africa conducted the interviews in French, which were in turn translated into the native Kirundi language by two local interpreters who had been trained in the relevant concepts of mental disorders. In order to standardize the form of assessment and to achieve a high inter-rater reliability, the interviewers practiced in joint interviews. To guarantee a precise translation, all instruments were translated from a validated English or French version to Kirundi and back into English or French by different interpreters and the results of the translation procedure were discussed in detail with the interpreters before the beginning of data collection. To guarantee confidentiality, it was assured that no other person was present or could listen to the interviews. The children were assured that everything they said during the interview was confidential and that there would be no negative consequences or punishment for whatever information was given. Furthermore, the children living in the center were given the opportunity to suggest improvements for the center. In addition to the interviews, the main researcher observed their behavior and performance in daily activities, school and joint playing. The study was part of a more comprehensive project that assessed violent behavior and the mental health situation of the children in residential care and of street children as part of overall consideration for possible interventions (Crombach and Elbert, 2014a,b).

### MATERIALS

#### Socio-demographics

The children were asked about their background and their actual social situation. This included particularly questions about their age and education. We also asked about their contact with the family, the age at which they left their family, as well as their reasons for doing so. Information on time spent in the streets and information about the whereabouts of their parents was also gathered.

#### Domestic and community violence checklist

This 37-item checklist assessed the children’s exposure to violence (following Hermenau et al., 2011). The events in the checklist range from small events like being pinched or slapped to very frightening events like being injured with a weapon or sexually abused. The checklist includes physical, psychological and sexual violence as well as neglect and witnessed violence. For every event the children were asked the following: If they were ever exposed to violence in their lives; if this happened regularly (at least 1/month in three succeeding months); or at least once over the past 3 months.

#### The University of California at Los Angeles PTSD Reaction Index (UCLA PTSD Index) for children and adolescents

The UCLA PTSD Index for children and adolescents (Steinberg et al., 2004) was used in interview form to assess the exposure to traumatic events and the severity of symptoms of PTSD. The latter is assessed based on the frequency of symptoms reported by children. The occurrence of each DSM-IV symptom within the last month is scored on a scale from none of the time (0) to most of the time (4). Thus an overall PTSD severity score can be calculated by summing up the symptom scores, which results in a maximum possible score of 68. A PTSD diagnosis was assumed if the DSM-IV criteria were fulfilled, including impairment in the daily functioning of the children in response to traumatic stress. The UCLA PTSD Index shows good psychometric properties and has been successfully utilized and validated in non-western and African settings (Shaw and Harris, 2003; Catani et al., 2008; Elbert et al., 2009; Hermenau et al., 2011). Inter-rater reliability was assessed by independently rating the same child in parallel, i.e., when both interviewers were present. The intra-class correlation of 0.99 (*p* < 0.001) indicated a high agreement among the interviewers.

#### Minnesota International Neuropsychiatric Interview Kid (M. I. N. I. Kid)

The sections A, C, J, and K of the MINI-KID (Sheehan et al., 2010) were used to assess depression, suicidal risk, alcohol and substance dependence or abuse. The MINI-KID has been used successfully in East-African settings (e.g., Hermenau et al., 2011). In order to allow a comparison between all children living in the center, the MINI-KID was used as well for the participants exceeding the age limit of 17 years. This seemed appropriate because all children and adolescents lived in the same conditions and still went to school.

### DATA ANALYSIS

The statistical analysis was carried out using SPSS 20.0 and AMOS 20.0 (IBM Corporation, Armonk, NY, USA). The hypothesis about group differences in PTSD severity and regularly experienced violence was tested calculating a multivariate analysis of variance (MANOVA). The effects of life within the center and violence on both PTSD and performance in school were analyzed via a path-model.

## RESULTS

### DESCRIPTION OF THE PARTICIPANTS

**Table 1** presents the demographic data of the 112 participants. The sample is divided into four categories: *former family children*, *former street children*, *street children,* and *family children*. As expected, the *street children* had spent significantly more time on the streets than the *former street children* within the center [*t*_(16.32)_ = -2.98, *p* = 0.009] and they had completed fewer school grades successfully than the other three groups (all *z* < -2.70, all *p* < 0.007).

**Table 1:** Demographic data.

	Former family children (*N* = 50)	Former street children (*N* = 32)	Street children (*N* = 15)	Family children (*N* = 15)
Age, years, mean (SD) [range]	15.5 (3.1) [11–23]	16.6 (2.6) [11–21]	16.2 (3.2) [12–24]	15.7 (3.1) [11–22]
Age, years, leaving family, mean (SD) [range]	11 (3.2) [5–17]	9.9 (2.7) [5–15]	10.4 (3.5) [4–16]	14 (0) [14]^b^
Time spent on the streets, months, mean (SD) [range]	0	18.7 (20.3) [0.1–84]	57.8 (48.9) [1.5–156]	0
Age, years, arriving in center, mean (SD) [range]	11.3 (3.4) [5–20]	11.5 (2.2) [7–17]	-^a^	-^a^
Time spent in the center, months, mean (SD) [range]	50.3 (26.4) [3–120]	60.3 (24.1) [7–120]	-^a^	-^a^
School grade successfully completed, mean (SD) [range]	5.6 (2.7) [0–11]	5.9 (2.2) [1–10]	3.0 (2.2) [0–8]	5.9 (2.8) [2–10]
Diagnose of, No (%) PTSD Major depression Alcohol dependence Alcohol abuse Substance dependence Substance abuse Moderate suicidal risk	7 (14) 3 (6) 0 (0) 2 (4) 0 (0) 0 (0) 0 (0)	8 (25) 2 (6.3) 1 (3.1) 1 (3.1) 0 (0) 0 (0) 2 (6.3)	7 (46.7) 0 (0) 1 (6.7) 0 (0) 9 (60) 1 (6) 2 (13.3)	1 (6.7) 0 (0) 0 (0) 0 (0) 0 (0) 0 (0) 0 (0)
Number of traumatic life events, mean (SD) [range]	4.7 (1.8) [1–9]	6.2 (2.0) [3–10]	7.0 (1.5) [4–9]	3.3 (2.8) [0–9]
Regularly experienced violence, No (%) Physical violence Sexual violence Witnessed violence Psychological violence Neglect	36 (72) 1 (2) 13 (26) 29 (58) 34 (68)	29 (90.6) 1 (3.1) 8 (25) 24 (75) 28 (87.5)	15 (100) 3 (20) 10 (66.7) 15 (100) 13 (86.7)	11 (73.3) 1 (6.7) 1 (6.7) 6 (40) 7 (46.7)

*–^a^Means that the question is not applicable to the group. ^b^Both family children lived with relatives in the capital, while their immediate family lived in the countryside.*

The prevalence of PTSD, depression, alcohol- and substance dependence/abuse and suicide risk (at least moderate) is presented in **Table 1**. Fisher’s exact tests, generalized for m × n tables (Mehta and Patel, 1983) showed that the frequency of substance dependency, in this case “chanvre,” a drug similar to marijuana, was higher in *street children* compared to the other groups (*p* < 0.001). The *street children* also had a higher prevalence of PTSD than the *family children* and the *former family children* (all *p* < 0.05). The frequencies of the other mental health disorders did not differ significantly between the groups.

Furthermore, the *number of traumatic life events* assessed with the UCLA PTSD Index is provided in **Table 1**. An ANOVA confirmed that on average *street children* and *former street children* did not differ regarding the number of experienced traumatic events (*p* = 0.67) and had been more exposed than the *former family children* and the *family children* (all *p* ≤ 0.05). By tendency, the latter group had been less exposed than the *former family children* (*p* = 0.06). To differentiate the kinds of violence, which the children had to face regularly throughout their lives, we decided to highlight the five prominent domains of the Domestic and Community Violence Checklist and to represent how many children of each category had been affected.

The most frequent reason children had for leaving their family was poverty (55%), followed by severe maltreatment (19%) within their families. Other reasons included being abandoned, having been forced to work very hard, death or divorce of parents and a variety of other reasons, including loss of contact with the family because of war, and searching for medical help. Some could not give a reason why the family had given the child to the center. In regard to the death or divorce of parents and family violence, the children often mentioned that the family did not want to have another boy around who could possibly become an heir. This was also reflected in the high percentage of orphans (33%) amongst the children living in the center and the street children. However most of the *former family children* (78%) and the *former street children* (69%) within the center and even of the *street children* (60%) have had at least some contact with their families over the last year. All children living in the center were either going to school or doing apprenticeship training. Not surprisingly, more than 90% of the *street children* had no access to education.

### PTSD SYMPTOM SEVERITY AND EXPOSURE TO VIOLENCE AND INSECURITY

**FIGURE 1:**
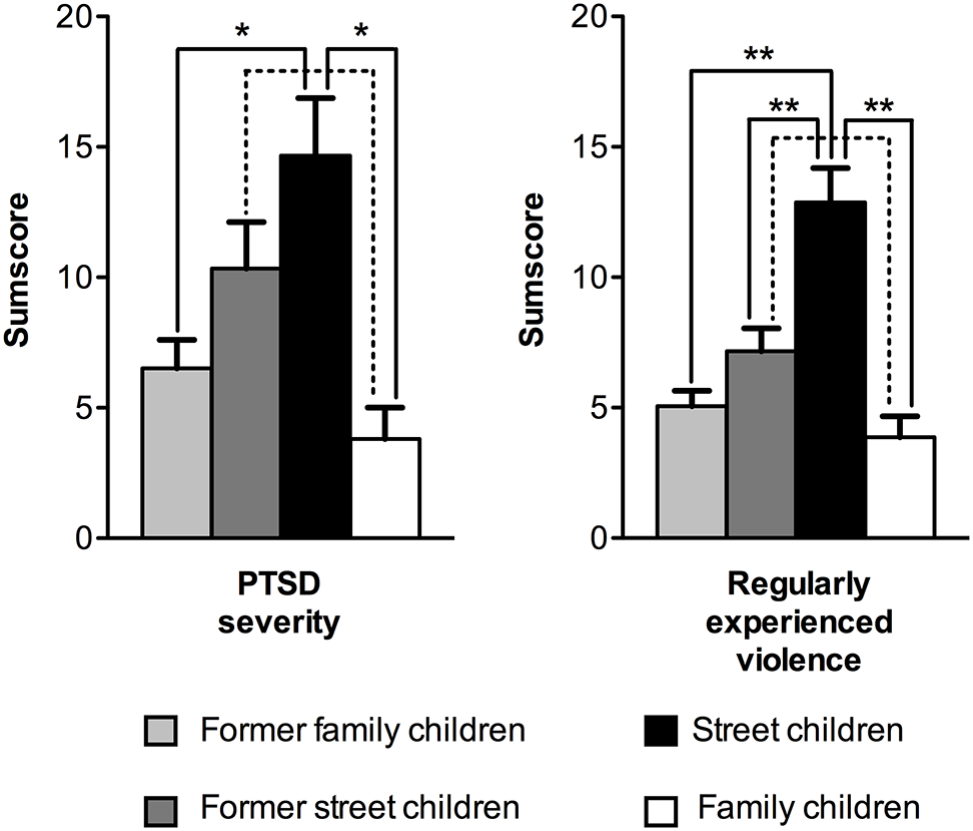
**Differences between the groups in *PTSD severity* (means and SEs) and *regularly experienced violence.*** Dotted lines (*p* ≤ 0.1), solid lines (*p* ≤ 0.05) one asterisk (*p ≤ 0.01) and two asterisks (**p ≤ 0.001) indicate the level of significance of the differences.

Our first goal was to assess the assumption that *street children* and *former street children* living in the center had been exposed more regularly to violence and insecurity over the course of their lives and suffered more from symptoms of PTSD. We calculated a MANOVA with the overall sum score of *PTSD severity* of the UCLA PTSD Index and the sum score of the *regularly experienced violence* of the Domestic and Community Violence Checklist. The statistical analysis revealed highly significant differences with moderate to strong effect sizes on the multivariate level [*F*_(6,214)_ = 7.05, *p* < 0.001, $\eta_{p}^{2}$ = 0.17], on the univariate level for the *PTSD severity* [*F*_(3,108)_ = 5.88, *p* = 0.001, $\eta_{p}^{2}$ = 0.14] and for the sum score of the *regularly experienced violence* [*F*_(3,108)_ = 14.09, *p* < 0.001, $\eta_{p}^{2}$ = 0.28]. Gabriel’s test was used for the *post hoc* tests because the group sizes differed (Field, 2009). As illustrated in **Figure 1**, the *post hoc* tests showed that the PTSD symptom severity was stronger for *street children* than for the *former family children* living in the center and for the *family children* (all *p* < 0.01). However they did not differ significantly from the *former street children* in the center (*p* = 0.43). The latter group did not differ significantly with respect to the *PTSD severity* from the *former family children* (*p* = 0.23) but did have somewhat more severe PTSD symptoms than the *family children* (*p* = 0.07). Concerning *regularly experienced violence*, the *street children* experienced more events than the *former family children,* the *former street children* and the *family children* (all *p* < 0.001). The three latter groups did not differ (all *p*> 0.05), even though the *former street children* showed a tendency toward having experienced more violence than the *family children* (*p* = 0.10). *PTSD severity* was strongly associated with both, the *number of traumatic life events* (*r* = 0.48, *p*≤ 0.001) and *regularly experienced violence* (*r* = 0.52, *p*≤ 0.001) within the total sample.

### MALTREATMENT, PTSD AND SCHOOL RESULTS

For the children living in the center we expected that their success in school – measured by the *number of classes successfully completed* – was not only influenced by the *time spent in the center*, the *age of arrival in the center* and the interaction between the two variables, but was also affected negatively by PTSD symptoms. Furthermore we predicted that the *PTSD severity* was not only determined by the exposure to the *number of traumatic life events* but also by the level of *violence experienced in the past 3 months*.

In order to test the above hypothesized relationships we conducted a path-analysis using AMOS 20 for SPSS. We used a backward stepwise method in a linear model until all beta coefficients were significant and the Akaike information criterion was lowest (see Akaike, 1987). The final model fitted the data according to the criteria for a good-fitting model and the combinatorial rules to reduce type I and type II errors (Hu and Bentler, 1999), χ^2^ (12) = 13.00, *p* = 0.369, χ^2^/df = 1.083, comparative fit index = 0.99, root-mean-square error of approximation = 0.03, *p* = 0.548. The model explained 75% of the variance of the *number of classes successfully completed* and 24% of variance of the *PTSD severity*.

**FIGURE 2:**
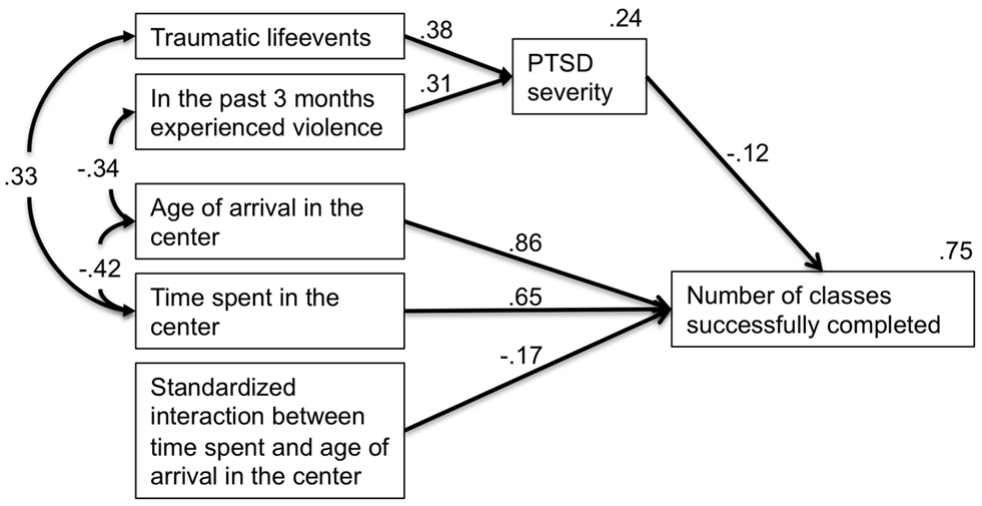
**Factors influencing level of PTSD symptoms and success in school in a center for vulnerable children**.

As can be seen in **Figure 2**, the *age of arrival in the center* and the *time spent in the center* positively predicted the *number of classes successfully completed*, indicating that if the children were older when they arrived in the center, they were more likely to be more advanced in school. Secondly, the more time they spent in the center, the more advanced they were likely to be in school. However, the interaction between the two variables negatively predicted the *number of classes successfully completed*, indicating that the older they were upon arrival and the longer they lived in the center, the less successful children became at school. As expected, the *PTSD severity* was positively predicted by the *number of traumatic life events* and also by the *violence experienced in the past 3 months*. The vast majority of these violent events – 81.62% – were minor physical conflicts, psychological violence or neglect. The *PTSD severity* had a negative influence on the *number of classes successfully completed*, underlining the assumption that suffering from PTSD is detrimental to success in school. The *age of arrival in the center* was negatively related to the amount of violence experienced in the past 3 months. The *time spent in the center* was positively correlated with experienced *traumatic life events*.

## DISCUSSION

### SUMMARY OF THE MAIN RESULTS

With this study we aimed to provide empirical data to strengthen the evidence of several hypotheses about trauma-related disorders in street children in post-conflict settings and thought to detail how to best assist the children in the reintegration process into society. In line with other studies (e.g., Stewart et al., 2004; Thomas de Benitez, 2007; Tyler et al., 2014) we found that in comparison to other vulnerable children, children who had spent parts of their lives in the streets had experienced greater levels of violence on a regular basis and suffered from stronger PTSD symptoms. Moreover, exposure to violence and levels of PTSD severity were correlated indicating that trauma-related mental disorders were more likely to occur in children who had encountered more violence and maltreatment. PTSD symptoms remained common among the former street children living a longer period of time within residential care highlighting the necessity to address trauma-related disorders with specific treatment approaches as part of the reintegration process. In contrast substance dependence was only common among current street children. Furthermore, we could provide evidence that recent maltreatment was associated with PTSD symptoms and that this had long-term effects upon progress in school. This result completes the findings of other studies in this field, showing that PTSD symptoms and exposure to traumatic violence lead to poor school performance (Elbert et al., 2009; Mathews et al., 2009; Catani et al., 2010).

### RESIDENTIAL CENTERS FOR STREET CHILDREN

Residential centers often fail to respond to the childrens’ psychosocial needs, especially those of the younger children, e.g., not sufficiently satisfying their attachment needs or providing sufficient help for a successful cultural and social integration in the society (Hermenau et al., 2011). However, living in the streets often exposes children and youths to high rates of violence, abuse and drug use, thereby endangering the development of the children (Thomas de Benitez, 2007; McManus and Thompson, 2008). Unfortunately the infrastructure for successful reintegration into a family system without abuse and maltreatment is often missing in the East African context and the process of reintegration is not as cheap and easy to accomplish as is often assumed. Residential centers are often the first line of defense in helping children living in the streets. These contradictions and the complexity of the situation were reflected in the results of this paper, which assesses the mental health of current street children and of children living in a residential center in Burundi.

In line with the criticism of Williamson and Greenberg (2010), we found that only about 33% of the children living within the center or on the streets could be strictly defined as orphans. In fact, most of the children had contact with a member of their family at least once a year. This was true for 69% of the former street children and 78% of the former family children living in the center, as well as for 60% of the children who were still living in the streets. Apparently, the percentage of children living in the center who had contact with their families was only slightly higher compared to the current street children. In a few cases, the lack of contact came about through a lack of knowledge of where to find the family. This separation was sometimes brought about by the war. However, in most cases it was either due to a lack of money for the journey home or the reluctance of the child to stay even a few days with the family. This was often out of fear of maltreatment and conflicts relating to private property inheritance. In some cases, children refused to return to their families because they were afraid of being forced to leave the center. This once again underlines the counter-productive results of educators using such threats as a means of gaining authority. It also demonstrates the necessity of creating a transparent and predictable procedure for reintegrating the children into their families.

However, before raising the argument that children who are in contact with their families should not be living in the center, we need to consider the fact that most of the children left their families because of severe poverty, which made it almost impossible to raise a child and/or lead to severe maltreatment within the family. Moreover, many children reported that in the case of death or divorce of parents, the family did not want to have another boy around who could possibly inherit. Further research is needed to evaluate when children and youths in Africa might be better off in residential centers and when they are better off staying with their own family. The present study focuses on the advantages of residential centers for extremely affected children only.

The results showed some of the advantages of living in residential centers. Firstly, all children in the center had access to education and, like children growing up in families, were more successful in school than children living in the streets. The most prevalent mental disorders in this sample were substance dependence and PTSD. While about 60% of the children living in the streets were affected by substance dependence, no child living in the center or in a family fulfilled these criteria. A possible explanation could be the combination of having less access to people selling drugs and having no funds to afford the drugs. The PTSD prevalence as well as symptom severity was different across groups. Children living in the streets had the highest prevalence and severity, followed by the former street children living in the center, the former family children living in the center, whilst the family children had the lowest prevalence and severity. Although this pattern was not statistically significant between all groups, it appears that the former street children fall between the street children and former family children in severity. A similar pattern could be seen in regularly experienced violence during the life of the children, indicating that the residential center offered at least some protection from violence experienced on a regular basis in the streets.

### NEEDS BEYOND FOOD, SHELTER, AND EDUCATION

The fact that the PTSD severity of the formerly exposed children within the center did not differ significantly from the children still living in the streets indicates that it is not enough to provide the children with a relatively secure environment with shelter, food and access to education. This is supported by the overall clinical impression that the children were extremely mistrustful and afraid, often feeling helpless and appearing to feel very easily threatened. In a path-model we further examined the relationship between the following variables: Firstly, the impact of the time the children spent within the center and the age of arrival in the center on both PTSD severity and school performance; secondly the model measured the impact of traumatic life events and violence upon both PTSD severity and school performance. This path-model revealed that the secure environment provided within the center was only relative. Even though the center was apparently protecting the children from being regularly exposed to violence, the positive relationship between the time spent within the center and traumatic life events indicates that there was a probability of experiencing very severe traumatic events within the center. This strong relationship most likely reflects a combination of factors. Some of the older children had experienced very dangerous and terrifying war events during the time they had spent at the center. Some of the children had been exposed to different forms of abuse within the center. The negative relationship between recently experienced violence and age of arrival in the center showed that the younger children in particular were exposed to further maltreatment within the center. This reflects the paradoxical situation in which children are taken from the streets as early as possible to protect them from regular exposure to violence, only to be again exposed to physical and psychological violence as well as neglect in a residential center.

The detrimental effects of maltreatment on the children can be seen in the path-model when examining how the success in school was negatively influenced by mental ill-health. The degree of PTSD severity had a negative impact on the performance in school over the years. The PTSD severity itself was not only influenced by the number of traumatic events but also by the exposure to recently experienced physical, psychological violence and neglect. The violence and neglect experienced, although not strictly “traumatic” in the sense defined for PTSD diagnosis, nevertheless creates a feeling of insecurity and helplessness that easily triggers the trauma-related fear network, provoking the related cognitions, emotions and physical reactions, thereby evoking strong feelings of present danger and fear within the individual. This reinforces PTSD symptoms and other trauma-related disorders and hence diminishes the functionality of the child. As most of this recently experienced violence consisted of either minor physical events like being slapped, psychological threats or neglect, these results again highlight how important a violence free upbringing is for children and how easily the fear network can be triggered within children who have lived through traumatic situations. Maltreatment of any kind is detrimental to the general mental well-being of children, and enhances the severity of PTSD symptoms (Catani et al., 2008). This effect is especially prominent in a post-conflict country like Burundi, where vulnerable children have been continually exposed to violence. Consequently, raising children in a residential center without strictly controlling violence directly undermines the objectives of such a center. It prevents children from successfully progressing in school or vocational training, thereby diminishing the chances of successful (re-) integration into society. The older the children were, when they arrived at the center, the less they were exposed to recent physical, psychological violence and neglect. This may be the reason why children arriving later at the center suffered less from PTSD, even though they had experienced more violence on a regular basis in their lifetime.

While PTSD symptoms had an effect on school results, the success in school was strongly influenced by the opportunity to go to school. Therefore it is not surprising that the time spent in the center predicted the number of successfully completed classes over the years. Interestingly, the older the children were when they arrived at the center, the more advanced they were in school. This effect is easily explained by the fact that not all the children lived in the streets. Some of them only arrived when the situation at home became intolerable, having already been in school. Moreover some of the children arriving later participated in a training program in order to start with the third class instead of the first class. The interaction between the time spent at the center and the age of arrival at the center suggests that the beneficial effect of living in the center is the strongest at the beginning, when the children arrive. The longer they stayed in the center the slower they progressed in school. This most likely reflects the fact that many children having faced hardship in their early years were unable to cope with the increasing demands of the higher classes.

### LIMITATIONS

The children in the center were afraid of being suddenly sent back to their families and of being left alone with their difficult life situation. Most of them had learned to be mistrustful of others, especially adults. Hence they were biased in reporting that everything was fine in order to protect their actual refuge. They had learned that it might be best to appear strong and keep quiet about problems. It is thus plausible to assume that the children in the center underreported traumatic stress. In fact, distrust in others and the reluctance to admit symptoms may be a manifestation of hyperarousal in traumatized youths (Whitbeck and Hoyt, 1999; Auerswald and Eyre, 2002; Baer et al., 2004). Furthermore, chronically traumatized persons and homeless youths are often ashamed of their problems and have a diminished understanding of self-care (Newman, 2000; Kidd and Kral, 2002). As the children and youths most likely experienced traumatic stressors during critical periods of their development, they may frequently be unable to access and regulate their emotional responding (Elbert et al., 2006). Therefore the effects of PTSD on success in school and the PTSD severity may actually be much stronger than they appear in this study. Observations during subsequent interventions and interviews conducted with the same children confirmed this impression.

## CONCLUSION

This study shows that residential centers can have beneficial effects for children who have lived in the streets, such as protecting them to a certain degree from constantly experiencing violence. However, all together the results strongly suggest that it is equally important to adhere to the psychological needs of vulnerable children as it is to satisfy other basic lacking needs in order to help them successfully reintegrate into society. The most important factor affecting their PTSD levels appears to be having a secure environment with no further physical violence, no acute threats and no ongoing neglect. Otherwise, a frequent activation, which leads to an enlargement of the associative fear network, will impair the functionality of the children. A well-trained and reliable team of educators may be the key to success here. Furthermore the effects of PTSD have again been demonstrated to reach beyond the realm of the child’s mental health, affecting school performance, and thereby the chances for successful reintegration into society (Elbert et al., 2009). As the former street children did not differ significantly from the current street children regarding PTSD symptom severity, a more targeted treatment of this mental disorder may be needed within residential centers.

### SUGGESTIONS FOR IMPROVEMENTS

Hermenau et al. (2011) pointed out there is very little research on what could be done to improve the situation in residential centers. Hence we want to stress the priorities that emerge from the results of the present study: a secure and predictable environment has to be established for the children. Children should be protected from violence, fear and helplessness. They should be motivated by reward rather than punishment. In the case of residential centers, a transparent structure has to be created and communicated, detailing when and how children are to be sent back to their families of origin. Since the final goal of residential centers is the successful reintegration of the children into society, frequent contact between children and family has to be a very high priority. The educators should have the opportunity to engage in activities with the children that allow them to bond and to help the children to establish trusting relationships.

Most importantly, the educators of these facilities need to be trained to avoid resorting to violence or threats as a means of control, because the effects of such responses are detrimental for the mental health of the children and destroy the efficiency of any aid. Many of the educators use violence or threats because they are not adequately prepared to cope with the challenges of a residential center. As Schauer and Schauer (2010) pointed out, there is a need for a paradigm shift in humanitarian aid for war-affected populations. Healing from trauma reduces emotional pain, decreases the likelihood of aggression and enables people to live productive lives. Hence an effective psychological treatment for PTSD ought to be provided for children within residential care.

## AUTHOR CONTRIBUTIONS

All authors developed the study concept and contributed to the study design. Anselm Crombach coordinated the project and performed a large number of the assessments. He performed the data analysis, the interpretation and drafted the paper. Manassé Bambonyé provided knowledgeable input regarding mental health projects within the Burundian culture and assisted overcoming administrative challenges. Thomas Elbert wrote the grant applications to achieve funding for this project. Furthermore, both provided critical revisions. All authors approved the final version of the paper for submission.

## Conflict of Interest Statement

The Reviewer, Sarah Wilker, declares that despite having collaborated with author Dr. Thomas Elbert, the review process was handled objectively and no conflict of interest exists. The authors declare that the research was conducted in the absence of any commercial or financial relationships that could be construed as a potential conflict of interest.
